# Three complete mitochondrial genomes of shortfin mako sharks, *Isurus oxyrinchus*, from the Atlantic and Pacific Oceans

**DOI:** 10.1080/23802359.2022.2060768

**Published:** 2022-04-11

**Authors:** Marissa R. Mehlrose, Andrea M. Bernard, Kimberly A. Finnegan, Lauren E. Krausfeldt, Jose V. Lopez, Mahmood S. Shivji

**Affiliations:** a Save Our Seas Foundation Shark Research Center, Nova Southeastern University, Fort Lauderdale, FL, USA; b Department of Biological Sciences, Halmos College of Arts and Sciences, Nova Southeastern University, Fort Lauderdale, FL, USA

**Keywords:** *Isurus oxyrinchus*, mitogenome, shortfin mako

## Abstract

We present complete mitogenome sequences of three shortfin mako sharks (*Isurus oxyrinchus)* sampled from the western Pacific, and eastern and western Atlantic oceans. Mitogenome sequence lengths ranged between 16,699 bp and 16,702 bp, and all three mitogenomes contained one non-coding control region, two rRNA genes, 22 tRNA genes, and 13 protein-coding genes. Comparative assessment of five mitogenomes from globally distributed shortfin makos (the current three and two previously published mitogenomes) yielded 98.4% identity, with the protein-coding genes ATP8, ATP6, and ND5 as the most variable regions (sequence identities of 96.4%, 96.5%, and 97.6%, respectively). These mitogenome sequences contribute resources for assessing the genetic population dynamics of this endangered oceanic apex predator.

The shortfin mako (*Isurus oxyrinchus;* Rafinesque, 1810, Lamnidae, Lamniformes), a highly migratory pelagic shark with a global distribution, is a species of strong conservation focus. This species is caught in large numbers worldwide and contributes significantly to the international shark fin trade (Clarke et al. 2006). Furthermore, fishing mortality for this species is higher than previously estimated in Atlantic stock assessments (Byrne et al. 2017). Given its dire population status, the shortfin mako is now classified as globally ‘Endangered’ (International Union for Conservation of Nature (IUCN) Red List; Rigby et al. 2019). Science-informed conservation management is urgently required for this species. Mitochondrial DNA partial control region sequence analysis suggests some global population structure may exist in this species despite its long-distance movements-based migratory lifestyle (Corrigan et al. 2018). Genome data will assist in fully resolving genetic population dynamics aspects of this endangered apex predator.

Fin tissues were obtained from three shortfin mako sharks sampled globally: (1) a shark of unknown sex captured in 2001 in the waters surrounding Whangarei, New Zealand (LAT: −35.72479°, LONG: 174.31572°) (NSU accession number OC-046; Mahmood S. Shivji, mahmood@nova.edu), (2) a male shark captured in 2009 in the eastern Atlantic (LAT: 20.00°, LONG: −23.50°) (NSU accession number OC-366; Mahmood S. Shivji, mahmood@nova.edu), and (3) a dead male whole shark carcass found drifted onto Dania Beach, Florida, USA in 2017 (LAT: 26.05395°, LONG: −80.11121°) (NSU accession number OC-398; Mahmood S. Shivji, mahmood@nova.edu). Sharks were identified to species morphologically, and the identification was confirmed via DNA barcoding (Cytochrome c oxidase subunit I). All tissues were stored in 100% ethanol, and DNA extraction was performed using the Qiagen DNeasy Blood and Tissue Kit (Qiagen Inc., USA) following the manufacturer’s protocol.

Individual shark mitogenomes were amplified in eight overlapping fragments using previously published primer pairs, as well as primers specifically designed for this study using sequences of two publicly available, shortfin mako mitogenomes [GenBank Accession Numbers: MF537044 (Gorman et al. 2019); KF361861 (Chang et al. 2015)]. Mitogenome libraries were generated using the Nextera XT DNA Sample Prep Kit (Illumina, CA, USA) and subsequently 2 × 250 PE sequenced on an Illumina MiSeq. Raw reads were quality filtered and trimmed using BBDuk 38.84 and assemblies were performed in duplicate using Geneious Prime 2021.2.2 (Biomatters, Aukland, NZ), the ‘Map to Reference’ feature, the Geneious Mapper tool, and both previously published mitogenomes as reference sequences. Mitogenome features were annotated using the program MitoAnnotator (Iwasaki et al. 2013) and verified against available shortfin mako DNA sequences. Inter- and intra-species sequence comparisons were performed via alignment (MUSCLE; maximum number of iterations = 8) of our three shark mitogenome sequences with the two previously published shortfin mako mitogenomes, as well as a single representative mitogenome sequence from each member of the family Lamnidae and the Lamniform outgroup, *Cetorhinus maximus*. A maximum likelihood phylogeny was constructed using MEGA 10.2.6 (Kumar et al. 2018) assuming the GTR + G substitution model with 1000 bootstrap iterations.

Our three shortfin mako mitogenome sequences were 16,699 bp (OC-398; gb: MZ923833; nucleotide composition: 28.9% A, 28% C, 15.1% G, and 28% T), 16,700 bp (OC-046; gb: MZ923831; 28.8% A, 28% C, 15.2% G, and 27.9% T), and 16,702 bp (OC-366; gb: MZ923832; 28.8% A, 28% C, 15.2% G, and 27.9% T) in length. Gene complement and order in our three mitogenome sequences were identical to the two previously published shortfin mako mitogenomes and consisted of two rRNA genes, 22 tRNA genes, 13 protein-coding genes, and one non-coding control region with a GC content of 43.2–43.3%. Percent sequence identity across all five shortfin mako mitogenomes was 98.4%; when samples were grouped according to ocean basin, percent identity was 99.7% for the two Pacific sequences and 98.6% for the three Atlantic sequences. Across all five mitogenomes, the highest variability was found in the protein-coding genes ATP8, ATP6 and ND5, with sequence identities of 96.4%, 96.5%, and 97.6%, respectively, which differs somewhat from Gorman et al. (2019) who reported the genes ND3, ND1 and ND5 as the most variable based on an assessment of two shortfin mako mitogenomes. Complete mitogenomic assessments of shortfin makos, rather than single region data analyses, may be more beneficial for deciphering nuanced genetic differences among populations and allow for more robust, data-driven management efforts to ensure the sustainability of this endangered oceanic predator (Figure 1).

**Figure 1.**
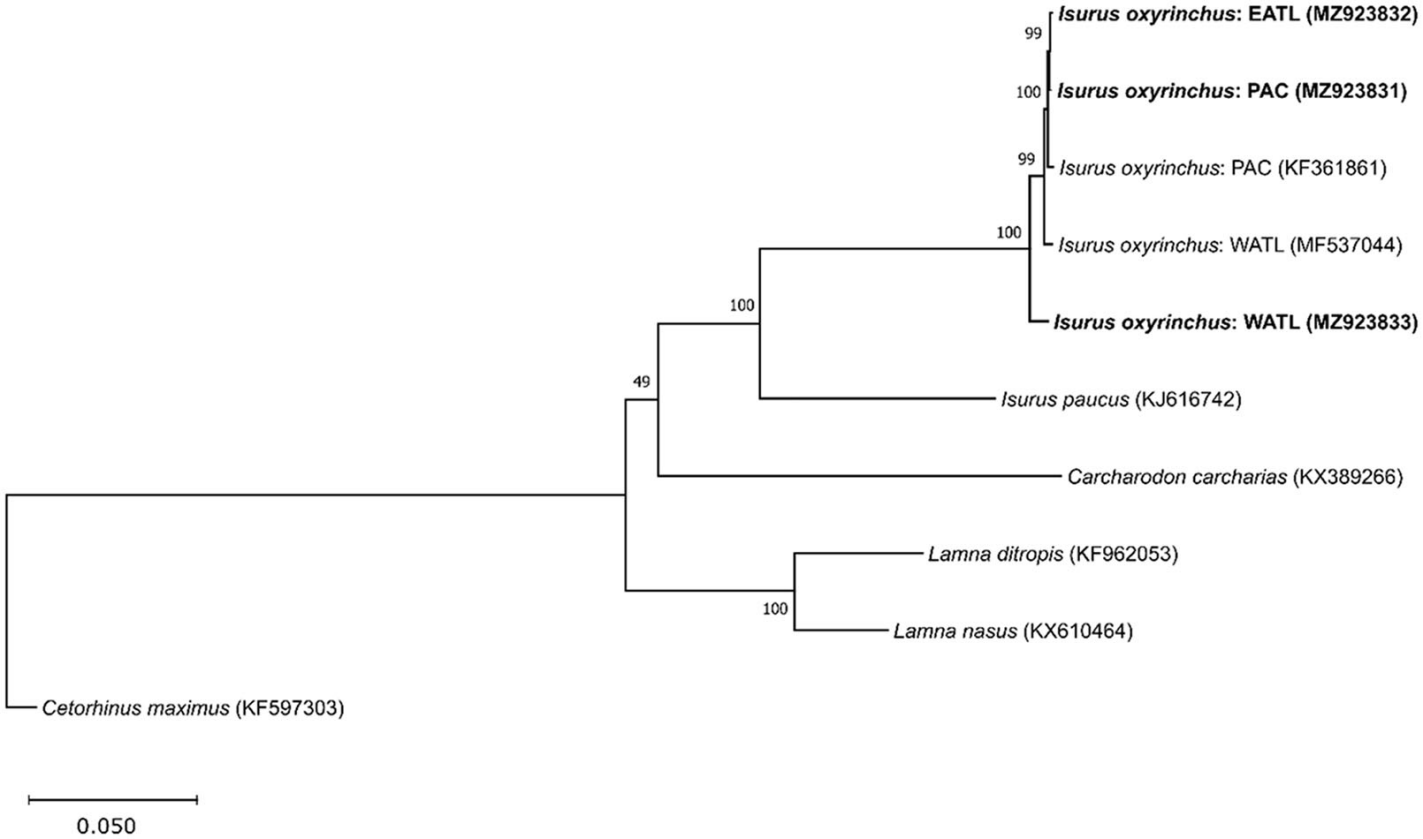
Maximum likelihood mitogenome phylogeny of the shark family Lamnidae, assuming *Cetorhinus maximus* (Cetorhinidae; Lamniformes) as the outgroup, the GTR + G model of evolution, and 1000 bootstrap replicates. The tree with the highest likelihood is shown with the percentage of cluster bootstrap support values. Shortfin mako geographic location abbreviations: EATL: eastern Atlantic Ocean; WATL: western Atlantic Ocean; PAC: Pacific Ocean. GenBank Accession numbers for each species and individual are shown in parentheses.

## Data Availability

The genome sequence data that support the findings of this study are openly available in GenBank of NCBI at https//www.ncbi.nlm.nih.gov under the accession no. MZ923831-MZ923833. The associated BioProject, SRA, and Bio-Sample numbers are PRJNA759903, SRR15702859-SRR15702861, and SAMN21208807-SAMN21208809, respectively.
